# Extracellular Vesicles: A New Frontier in Biomarker Discovery for Non-Alcoholic Fatty Liver Disease

**DOI:** 10.3390/ijms17030376

**Published:** 2016-03-14

**Authors:** Linda A. Ban, Nicholas A. Shackel, Susan V. McLennan

**Affiliations:** 1 Greg Brown Diabetes and Endocrine Laboratory, Charles Perkins Centre, University of Sydney, NSW 2006, Australia; linda.ban@sydney.edu.au; 2 Liver Cell Biology Laboratory, Centenary Institute of Cancer Medicine and Cell Biology, Camperdown, NSW 2006, Australia; n.shackel@centenary.usyd.edu.au

**Keywords:** biomarkers, diagnosis, exosomes, extracellular vesicles, microvesicles, NAFLD, non-alcoholic steatohepatitis (NASH), steatosis, steatohepatitis

## Abstract

In recent years, the global burden of obesity and diabetes has seen a parallel rise in other metabolic complications, such as non-alcoholic fatty liver disease (NAFLD). This condition, once thought to be a benign accumulation of hepatic fat, is now recognized as a serious and prevalent disorder that is conducive to inflammation and fibrosis. Despite the rising incidence of NAFLD, there is currently no reliable method for its diagnosis or staging besides the highly invasive tissue biopsy. This limitation has resulted in the study of novel circulating markers as potential candidates, one of the most popular being extracellular vesicles (EVs). These submicron membrane-bound structures are secreted from stressed and activated cells, or are formed during apoptosis, and are known to be involved in intercellular communication. The cargo of EVs depends upon the parent cell and has been shown to be changed in disease, as is their abundance in the circulation. The role of EVs in immunity and epigenetic regulation is widely attested, and studies showing a correlation with disease severity have made these structures a favorable target for diagnostic as well as therapeutic purposes. This review will highlight the research that is available on EVs in the context of NAFLD, the current limitations, and projections for their future utility in a clinical setting.

## 1. Introduction

Obesity is rapidly evolving into a global pandemic, and poses a significant healthcare and socioeconomic burden. Its increased prevalence in both developed and developing nations has seen a rise in other serious metabolic complications, such as cardiovascular disease, type 2 diabetes mellitus and non-alcoholic fatty liver disease (NAFLD). Although diabetes is a common risk factor for NAFLD progression and *vice versa* [1,2,3,4], lean or non-diabetic patients also develop NAFLD [5,6,7], and so biochemical rather than anthropometric parameters would likely be of greater utility in diagnosis or prognosis of the disease.

To address this issue, the World Gastroenterology Organisation (WGO) recently published a set of comprehensive guidelines on the assessment and management of NAFLD [8], with emphasis on the distinction between simple steatosis and non-alcoholic steatohepatitis (NASH). The latter represents the advanced manifestation of the NAFLD spectrum whereby inflammation and fibrosis are also present, and is a condition which is much easier to identify than simple steatosis. However, limitations with current diagnostic methods, such as unreliable imaging techniques and serum markers, have meant that tissue biopsy remains the gold standard for NASH diagnosis [9,10,11,12,13,14]. Irrespective of this, biopsy is a highly invasive procedure and subject to variability through sampling error [15,16,17]. Moreover, it cannot predict disease progression, and, for this reason, there is increasing emphasis on the identification of stable non-invasive markers specific for liver disease progression.

At this stage, effective early detection is poor as patients usually do not report symptoms until they have progressed to NASH or cirrhosis. Serum biochemistry that reveals elevated liver transaminases in the absence of excessive alcohol consumption or other liver disease is the most typical indicator of NAFLD, while anthropometric data such as a high body mass index (considered obese if above 35 kg/m^2^) may warrant further screening for visceral fat accumulation in the liver [8]. It must nonetheless be stressed that despite the increased likelihood, not all obese individuals will develop NAFLD/NASH, and so probing for markers of steatosis in global metabolic disorders should therefore address what is known about the mechanisms of disease within the target organ. Ideal marker candidates should reflect not only the presence of NAFLD, but also the severity of disease, which is vital for early diagnosis as well grading progression [13].

This review aims to introduce the concept of using circulating cell-derived vesicles as novel markers of NAFLD, with an emphasis on their role in diagnosis and the assessment of disease pathology. Drawing on recent evidence from the literature, the paradigm of “marker *versus* mediator” will be discussed, as well as insight into their potential as therapeutic targets.

## 2. Novel Biomarkers in Liver Disease

In the latter half of the last century, shedding of vesicles from the cell membrane was identified as an inconsequential by-product of cell degradation [18,19]. However, clinical studies supported by research findings have recently pointed to the regulated secretion of these extracellular vesicles and their role in intercellular communication. Moreover, the abundance as well as the phenotype of circulating vesicles is reported to change in many disease states, including liver diseases [20,21,22,23] and metabolic disorders such as diabetes and obesity [24,25,26,27]. As such, much interest has been invested in characterising these structures for their potential utility in diagnostics, especially for conditions where this is otherwise notoriously difficult, such as NAFLD.

### 2.1. Extracellular Vesicles: What Are They?

**Figure 1:**
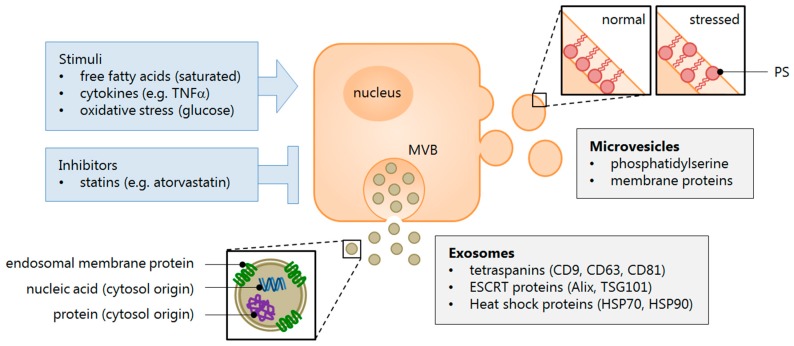
Extracellular vesicle characterisation. Cells respond to a variety of stimuli that cause inflammation and metabolic stress, which result in their activation, impaired functioning, or apoptosis. This mechanism drives the release of extracellular vesicles (EVs), which signal to paracrine or distal effectors the condition of the cell microenvironment. Effector cells may, in turn, respond by selectively imparting regulatory molecules—small nucleic acids (mRNA and miRNA), lipids, and proteins—contained within EVs, that are taken up by the recipient cell. The EV subclasses are identified by membrane markers that denote the site of their biogenesis. Exosomes typically express endosomal membrane proteins, such as tetraspanins, while microvesicles are understood to contain phosphatidylserine. These lipoproteins are normally oriented towards the cytosol to maintain the cell membrane asymmetry, but during conditions that stimulate EV release, the molecules become everted. Abbreviations: ESCRT = endosomal sorting complex required for transport; MVB = multivesicular body; PS = phosphatidylserine.

Extracellular vesicles (EVs) are collectively represented by three subclasses of membrane-bound structures that are distinguished based on their size, typical markers, and biogenesis [28,29,30] (see Figure 1). Exosomes are the smallest vesicles, usually below 100 nm in diameter, and are formed within multivesicular bodies (MVB) that release their contents into the interstitium upon fusion with the cell membrane. These exocytosed EVs are characterised by their expression of membrane tetraspanins, most notably CD63, as well as the endosomal sorting complex required for transport (ESCRT)-associated protein Alix, both of which reflect the MVB origin of exosomes [29,31,32].

In contrast, microvesicles (MVs) are shed directly from the cell membrane by a “budding” process and typically range in size from around 100 to 1000 nm, although these values are somewhat arbitrary and subclass overlap may exist [29]. MVs are identified by the expression of phosphatidylserine (PS) on their surface, which is indicative of their release from activated or apoptotic cells. In these cells PS is externalized, whereas in quiescent cells the membrane PS has a cytosolic orientation [33,34]. Most studies utilise the fact that Annexin V—a soluble protein used in the detection of apoptotic cells—binds with high affinity to PS and is therefore a useful marker of the MV subclass. Meanwhile, some groups have argued that a majority of circulating MVs are in fact PS-negative, whilst others have proposed that measurement of lactadherin may be a more sensitive alternative to Annexin V [35,36,37]. Despite ongoing controversies in their characterisation, both EV populations have ultimately been shown to impart functional properties of their parent cells through the transfer of proteins, mRNAs, and particularly microRNAs (or miRNAs) that are subsequently involved in epigenetic regulation [38,39].

Finally, apoptotic bodies represent the largest EV subclass in terms of their size, ranging from one to four microns. Since this is comparable with platelets, studies that use size exclusion techniques to isolate circulating EVs, such as ultracentrifugation or filtration, will usually lose this population of vesicles with larger contaminants [40]. Furthermore, as apoptotic bodies are formed during the compartmentalization of apoptotic cells, they are generally assumed to be inert particles destined for phagocytosis, although their horizontal gene-transfer capacity has been documented [41,42].

### 2.2. Role of Extracellular Vesicles in Liver Disease

Almost all cell types ubiquitously release low levels of extracellular vesicles. In normal physiology, most circulating EVs are derived from platelets and endothelial cells, and have been shown to be important in common haemostatic events such as coagulation [43]. While vesicles of the same origin have been implicated in disease complications of a pro-coagulative nature [44,45], there is still a paucity of knowledge regarding the dynamics of EV secretion by different cell types and in particular how the secreted EVs interact to advance the pathogenesis of a given disease. Controlled *in vitro* experiments have provided the most direct lines of evidence for EV regulation, including how the stimulus for release may affect their phenotype [46]. There is a wealth of research using liver injury models to explore EV-mediated fibrosis [47,48,49], transcriptomic signalling [50,51,52,53,54], and targeted immunotherapy [55,56,57] in artificial cell culture systems. However, *in vivo* studies present an added degree of complexity due to the difficulty of identifying liver specific EVs within the circulating pool. For this reason, most studies have opted to focus on circulating vesicle characterisation and their temporal changes in relation to liver disease development [58,59,60,61,62,63], while others have pointed to roles in extrahepatic cancer metastasis to the liver [64,65,66], although functional relationships have yet to be explored.

Some groups have approached the study of EVs from a more organ-targeted perspective, assessing their role as paracrine mediators. Most of these studies evaluate the effect of EVs in fibrogenesis, for example, the shuttling of pro-fibrogenic connective tissue growth factor (CTGF) between hepatic stellate cells on the one hand [47], or the CTGF inhibiting miRNA-214 between stellate cells and hepatocytes or adjacent stellate cells on the other hand [48]. Immune-mediated modulation has also been suggested; one study had demonstrated a role for T cell-derived EVs in the induction of stellate cell fibrolytic activity, as defined by an increase in the gene expression of matrix metalloproteinases (MMPs) [49]. The findings concluded that this response from the stellate cells was likely mediated by the homodimeric interaction of CD147 at the EV-cell interface. A pro-inflammatory glycoprotein, CD147 had previously been implicated in liver disease pathogenesis by our group [67,68] as well as having a well document role in tumour metastasis, which more recently had been attributed to EV-mediated translocation [69,70,71]. Secreted vesicles have also been linked to paracrine signalling in the tumour microenvironment, whereby miRNAs shuttled from hepatoma cells were able to modulate protein expression in adjacent hepatocytes and to increase their proliferative potential [50,51]. Silencing of these miRNAs, in turn, had abrogated the pro-tumorigenic effects, while another study had suggested a role for liver stem cell-derived EVs in miRNA-mediated tumour suppression [52].

### 2.3. Markers or Mediators of Liver Disease?

Taken together, this body of evidence highlights the growing expanse of EV research pertaining to liver disease, and on the contrary, a relative paucity of data regarding the involvement of EVs in NAFLD progression to NASH. Additionally, it introduces the “marker *versus* mediator” paradigm when addressing the functionality of EVs. This plays an important role in EV analysis; for instance, in the context of NAFLD, global changes in the circulating pool (marker) may not reflect the local interactions within specific tissues, such as the liver, that drive pathogenesis at these sites (mediator). However, a circulating profile that is unique to a given disease etiology would still substantiate the use of EVs as non-invasive diagnostic markers, a concept that is discussed further in the section below.

## 3. Studies in Non-Alcoholic Fatty Liver Disease

**Figure 2:**
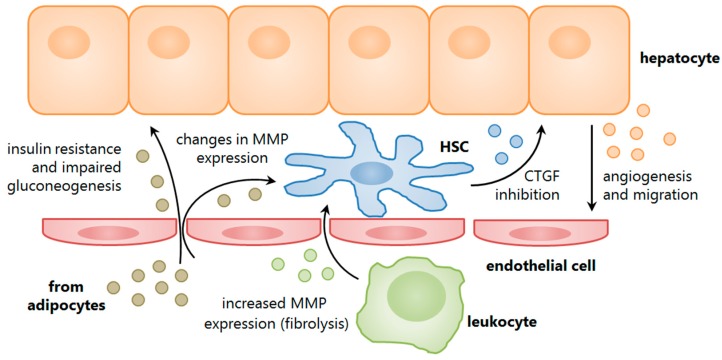
Extracellular vesicle roles in non-alcoholic fatty liver disease (NAFLD). EVs are involved in intercellular communication within the liver tissue, between hepatic cells as well as other tissues involved in mediating NAFLD pathogenesis, such as adipose and circulating (liver-homing) leukocytes. Collectively, these EVs are involved in a dynamic response that may exacerbate tissue injury, as well as promoting repair and matrix remodelling. Abbreviations: CTGF = connective tissue growth factor; HSC = hepatic stellate cell; MMP = matrix metalloproteinase.

Liver research involving EVs as disease mediators faces a number of inherent challenges. The most important of these is finding a link between the circulating EV populations and a specific contribution from the liver. From a biomarker perspective, it could be argued that a quantitative or phenotypic change in circulating EVs with disease may validate their diagnostic utility, especially if these changes are intensified with NAFLD progression (see Table 1). Unfortunately, given the complex biological determinants of EV secretion, rather than a linear relationship we are more likely to see dynamic responses from different tissues during the course of pathogenesis (see Figure 2). For a start, NAFLD is not an isolated condition and, generally speaking, occurs as a complication of other metabolic disorders where global insulin resistance is also present. Therefore, multiple tissues may be affected by the resulting oxidative stress and fatty acid flux, which in turn promotes the activation of immune cells and their migration to these sites. Consequently, the extrahepatic release of EVs may in fact mask the pathogenesis of NAFLD. For this reason, and the lack of a specifically hepatic molecular marker, ideal studies should examine the circulating EVs against their liver-derived counterparts, where possible.

### 3.1. Animal Studies

The fact that such issues remain to be addressed can be explained by the relative infancy of this field of research. To date, there are fewer than a dozen studies to have documented a role for EV signalling in a model of NAFLD, the earliest reported as late as 2009 in mice [79]. To better define a role for EVs in the development of hepatic steatosis, researchers have sought to replicate the clinical observations in rodent models of NAFLD, simulated by administering a choline-deficient diet (CDD) or high-fat diet (HFD) *ad libitum* for several weeks, the latter of which more accurately reflects the development of human metabolic syndrome. It should also be noted, that while CDD animals have comparable liver triglycerides to HFD animals, and a much more rapid progression to hepatic fibrosis, other typical changes such as increased body weight and fat depots, insulin resistance, and elevated fasting glucose and fatty acids are not observed [80]. This is due to the fact that, while HFD feeding increases lipid production, choline deficiency results in mitochondrial dysfunction and hence prevents the normal breakdown of lipids [81]. In saying that, contrary to what would be expected, EV studies in rodent models of NAFLD showed similar trends for both diets (see Table 2).

In the original study, Deng and colleagues described a phenomenon in their chronic HFD model whereby circulating EVs that were adoptively transferred to healthy animals were engulfed by myeloid cells that subsequently accumulated in the liver [79]. This phenotype was not observed when EVs were transferred from animals on a normal chow diet, which may suggest a selective, EV-driven mechanism for hepatic inflammation as a concomitant to steatosis. While these findings are yet to be reproduced, other groups have instead begun to more comprehensively examine the profile of circulating EVs to better understand their temporal regulation, contents, and possible intervention strategies. Indeed it was shown that vesicles tend to increase on a background of NAFLD, and do so in a time-dependent manner, according to data obtained from flow cytometry experiments [74,75,76].

To evaluate how the liver contributes to this population, EVs were assessed for their expression of miRNA-122, a molecule that is enriched in mammalian livers and is shown to be involved in early NAFLD progression [83,84,85]. Consistent with previous findings, rodent studies confirmed an increase in circulating EV-associated miRNA-122 accompanied by a decrease in the liver expression of this molecule [75,76,77]. Furthermore, one study demonstrated that when miRNA-122 was trafficked in EVs, it was not associated with its protein binding partner Argonaute 2, a phenomenon that is otherwise typically observed in non-disease conditions [75]. While other miRNAs and proteins were not correlated against disease severity, Povero and colleagues had employed mass spectrometry to identify an EV-specific proteome in NAFLD that was distinct from healthy controls [75]. These findings complement a previous study done by the group, in which they confirm a role for EV-bound Vanin-1 in hepatocyte vesicle uptake by an endothelial cell line, with subsequent angiogenic behaviour that is only observed when EVs are derived from hepatocytes subjected to lipotoxic stress [76].

Taken together, these studies establish a solid foundation for understanding the role of EVs in NAFLD, however, some notable limitations exist. Firstly, changes in EV phenotype were not correlated against histological severity of liver disease, which would otherwise give some insight into their prognostic value. Furthermore, perhaps an emphasis on distinguishing NAFLD from other underlying liver pathologies would give EVs a stronger diagnostic utility, as had been addressed in the clinical studies below.

### 3.2. Human Studies

The pioneering study to involve human subjects was published three years later by Kornek and colleagues, who for the first time had suggested a correlation between the circulating abundance of leukocyte-derived EVs and disease severity, as determined by liver transaminase levels, biopsy grade, and NAFLD activity score (NAS) [72]. These findings still provide the most compelling evidence in clinical samples for the prognostic value of EVs in NASH development, and have been extensively cited. The authors have additionally noted a distinction between the circulating NAFLD EV profile and that seen in hepatitis C patients. This is further supported by another study where transcriptomic analysis revealed that serum exosome-derived miRNAs are capable of differentiating multiple aetiologies of liver disease, as well as disease from normal liver controls [82]. Similar to the first study, it was shown that the expression level of some miRNAs was regulated either positively or negatively with histological features of disease, such as inflammation and fibrosis. However, these results were limited to the cohort with chronic hepatitis and no such data was available for NAFLD progression to NASH.

More recent studies have described the modulation of hepatocyte and stellate cell activity by EVs isolated from visceral (peritoneal) adipose tissue. While the subjects did not necessarily present with NAFLD, the *ex vivo* experimental designs instead aimed to establish a role for EVs in potentially mediating this disease. As such, Kranendonk and colleagues showed that adipocyte EVs from non-obese patients were capable of interfering with insulin signalling and gluconeogenesis when directly exposed to a hepatocyte cell line [78]. Furthermore, the concentration of EVs correlated positively with expression of liver transaminases, which supports the evidence for their role in hepatocyte dysfunction. In another study, albeit on a smaller scale, adipose tissue isolated from obese patients released EVs in culture that subsequently altered the gene expression of an MMP inhibitor, TIMP-1, in both hepatocytes and stellate cells [86]. Collectively, these findings suggest a novel mechanism of NAFLD pathogenesis by EVs through adipocyte-mediated hepatic cell stress and tissue remodelling.

## 4. Understanding the Role of Secreted Vesicles

With the urgency to develop a non-invasive biomarker for the diagnosis and staging of NAFLD, research into the biology of extracellular vesicles has provided an opportunity to explore a novel mechanism of disease pathogenesis that can also be harnessed as a clinical tool. However, there is still a long way to go before EV-related assays will have translational utility. Besides the obvious question of disease and tissue specificity, current techniques used in the isolation and characterisation of EVs remain laborious, and suffer from a lack of standardization, as well as high variability. It will undoubtedly take a few years before the processing of EVs from blood and other bodily fluids as “liquid biopsies” becomes economically viable, reproducible and validated. Until then we are unlikely to see their use in routine clinical practice.

While much can be learned from the studies described in this review, the concept of analysing EVs in the context of NAFLD is still very much a small niche in the literature. One reason could be the limitations mentioned above, or a focus on more accessible biochemistries such as liver transaminases and soluble miRNA-122. But then why look at circulating EVs? Perhaps the answer lies in their active role in disease; they may not only confirm the presence of NAFLD, but also give an insight into which tissues are interacting and how this is driving pathogenesis. It has been shown that adipose tissue EVs taken from obese individuals are capable of signalling to hepatic cells to remodel their extracellular milieu, while these cells in turn may communicate via EVs with the sinusoid to promote angiogenesis [76,86]. Circulating vesicles have also been implicated in the innate immune response that accompanies steatosis, pointing to a role in the progression from early NAFLD to NASH [72,79]. From a physiological perspective, it makes sense to encapsulate certain molecules that are otherwise prone to enzymatic degradation, especially in a complex or unpredictable disease environment. However, if preservation of these molecules within EVs leads to a heightened stimulation of inflammatory cells, as previously suggested, this mechanism may in turn be responsible for the exacerbation of tissue injury.

Whether EVs can be considered as friend or foe in metabolic diseases is still a grey area, and likely depends on the tissue of origin. Their use as a biomarker is further complicated by the possibility of temporal fluctuation or waning, as is seen with liver enzymes in models of NAFLD [87,88], which limits their predictive value. Furthermore, high-powered micrographs of liver sections have shown that hepatic EVs are predominantly located in the perisinusoidal region [75,76], which may indicate their entrapment in the liver, contrary to previous findings described in this review and also within the same studies. This idea is supported by the fact that the sinusoidal endothelium undergoes defenestration with progressive fibrosis, as well as aging [89], which may restrict the normal flux of vesicles and macromolecules within the liver. Alternatively, the accumulation of fibrous tissue in the perisinusoid may also limit the passage of EVs, or provide selective permeability to smaller vesicles. However, whether this is a protective mechanism or passive consequence of disease is yet to be elucidated.

### What Does the Future Hold?

The multifaceted nature of EVs suggests that these structures may have potential value beyond their use as circulating biomarkers in NAFLD. For instance, cancer studies have explored the transfer of oncogenes and an oncogenic phenotype through EV uptake in cell culture models [41,90,91], which may provide a target for therapeutic intervention. Indeed, it was shown that incubating hepatoma cells with various anti-cancer drugs promoted the secretion of immunogenic EVs that were capable of enhancing natural killer (NK) cell responses [55,56]. Conversely, exposing macrophages to such drugs may induce the release of EV-derived miRNAs, which suppress cancer growth by epigenetic regulation [57]. This concept has been extended to NAFLD models, where it was found that administering cholesterol-lowering drugs to high-fat fed rodents can attenuate the release of EVs, however the exact implication of this was not discussed, except for a potential reduction in liver cell death [73,74].

Another approach is to use the vesicles themselves as a mode or target of therapy, not simply a marker of injury. This idea has been investigated since the late 1980s, whereby synthetic EVs were used as a vehicle for drug delivery in both *in vitro* and *in vivo* models of liver injury [92,93]. It is also possible that in the future, endogenous EVs may be harvested for similar purposes, providing an efficient technique for tissue-specific delivery of molecules. The advantage of this autologous transfer system is that the vesicles are less likely to be rejected by the patient, however still sufficiently immunogenic to elicit a response [79].

## 5. Conclusions

With the rapid advancement of technology, it can be expected that once EVs become a routine parameter for assessment of disease status—of especial value in conditions that are difficult to diagnose, such as NAFLD—their utility may be further projected to the treatment of disease in its early stages, and potentially the reversal of chronic disorders like NASH. While there is still a long way to go, for the time being it is important to focus on controlling the underlying metabolic disorders through traditional intervention methods and lifestyle changes, which would also slow the progression of its comorbidities. However, detection of NAFLD and its staging continues to be a problem with invasive techniques such as biopsy being the gold standard. For this reason, EV analysis has promise as a non-invasive diagnostic tool.

## Figures and Tables

**Table 1:** Extracellular vesicle markers in non-alcoholic fatty liver disease (NAFLD) studies.

Vesicle Source		Marker(s)	Key Study Findings	Citation
Circulating	Lymphoid cells	CD4 CD8 Va24/Vb11	Enriched in NAFLD, positively correlated with serum ALT and liver biopsy	[72]
	Myeloid cells	CD14 CD15	Variable; CD14^+^ (monocyte origin) enriched in NAFLD, positively correlated with serum ALT; CD15^+^ (neutrophil origin) opposite trend	[72]
	Erythrocytes	TER119	Comprise the majority of circulating EVs during Western diet	[73]
	Platelets	CD41 CD62P	Conflicting data for abundance in NAFLD; reduced with statin intervention	[72,74]
	Liver	ASGPR1 CES1 miR-122 miR-192	Enriched in NAFLD; miR-122 and miR-192 correlated with decreased liver expression	[75,76,77]
	Endothelial	CD144	Enriched in NAFLD; reduced with statin intervention	[74]
Tissue derived	Adipose	adiponectin IL-6 MCP-1 MIF	Enriched in adipose origin; with the exception of adiponectin, enriched in visceral *versus* subcutaneous adipose	[78]
	Hepatocytes	Vanin-1	Enriched in steatotic hepatocytes (HepG2 cells treated with palmitate)	[76]

Abbreviations: ALT = alanine transaminase; ASGPR1 = asialoglycoprotein receptor 1; CES1 = (liver) carboxylesterase 1; IL-6 = interleukin 6; iNKT = invariant natural killer T [cell]; MCP-1 = monocyte chemotactic protein 1; MIF = (macrophage) migration inhibitory factor; NAFLD = non-alcoholic fatty liver disease; Va24/Vb11 = T cell receptor covariants a24/b11.

**Table 2:** Important findings for extracellular vesicles in the context of NAFLD.

Key Study Findings		Disease Model	Vesicle Source	Methods	Citation
Rodent	NAFLD-inducing diet increases circulating EV abundance	HFD CDD	plasma	FC	[74,75,76]
	Circulating EV abundance correlates with NAFLD progression	CDD	plasma	FC	[75,76]
	NAFLD-inducing diet increases circulating liver-derived EVs	HFD CDD	plasma serum	RT-qPCR	[75,76,77]
	NAFLD-inducing diet changes circulating EV contents	CDD	plasma	LCMS WB	[75,76]
	NAFLD-inducing diet changes circulating EV interactions with cells	HFD	plasma	FC	[79]
Human	Circulating EV abundance correlates with NAFLD progression	NASH	plasma	FC	[72]
	Circulating EV contents can distinguish NAFLD from other liver diseases	NASH	plasma serum	FC microarray	[72,82]

Abbreviations: CDD = choline deficient diet, EV = extracellular vesicle, FC = flow cytometry, HFD = high-fat diet, LCMS = liquid chromatography with mass spectrometry, NAFLD = non-alcoholic fatty liver disease, NASH = non-alcoholic steatohepatitis, RT-qPCR = real-time quantitative polymerase chain reaction, WB = western blot.

## Abbreviations

The following abbreviations are used in this manuscript:

ALT: alanine transaminase
ASGPR1: asialoglycoprotein receptor 1
CDD: choline-deficient diet
CES1: carboxylesterase 1
CTGF: connective tissue growth factor
ESCRT: endosomal sorting complex required for transport
EV: extracellular vesicle
FC: flow cytometry
HFD: high-fat diet
HSC: hepatic stellate cell
IL-6: interleukin 6
iNKT cell: invariant natural killer T cell
LC-MS/MS: liquid chromatography with tandem mass spectrometry
MCP-1: monocyte chemotactic protein 1
MIF: [macrophage] migration inhibitory factor
miRNA: microRNA
MMP: matrix metalloproteinase
MV: microvesicle
MVB: multivesicular body
NAFLD: non-alcoholic fatty liver disease
NAS: NAFLD activity score
NASH: non-alcoholic steatohepatitis
NK cell: natural killer cell
PS: phosphatidylserine
RT-qPCR: real-time quantitative polymerase chain reaction
TEM: transmission electron microscopy
TIMP-1: tissue inhibitor of metalloproteinase 1
WB: western blot
WGO: World Gastroenterology Organisation
